# Three-dimensional morphometric differences of resected distal femurs and proximal tibias in osteoarthritic and normal knees

**DOI:** 10.1186/s12891-021-04889-z

**Published:** 2021-12-04

**Authors:** Xiang-hui Dong, Xiang-hui Huang, Ming Chen, Yan-Hai Chang, Ming Ling, Bo Yang

**Affiliations:** 1 Department of Orthopaedics, Shaanxi Provincial People’s Hospital (third affiliated hospital of Xi’an jiaotong University), No. 256, Youyi West Road, Xi’an, China; 2 Shaanxi Provincial Key Laboratory of Infection and Immune Diseases, Shaanxi Provincial People’s Hospital (third affiliated hospital of Xi’an jiaotong University), No. 256, Youyi West Road, Xi’an, China

**Keywords:** Computed tomography, Osteoarthritis, Normal knee, Total knee arthroplasty, Morphometry

## Abstract

**Background:**

There is a paucity of data concerning the morphological differences of resected distal femurs and proximal tibias in osteoarthritic (OA) and normal knees. The objective of this study was to determine whether morphometric differences in the surfaces of resected distal femurs and proximal tibias exist between OA and normal knees in a Chinese population.

**Methods:**

Ninety-four OA knees and ninety-five normal knees were evaluated in Chinese individuals. Computed tomography was used to measure the femoral mediolateral (fML), medial anteroposterior (fMAP), lateral anteroposterior (fLAP), medial condylar width (fMCW), lateral condylar width (fLCW), medial posterior condylar curvature radii (fMCR), lateral posterior condyle curvature radii (fLCR), fML/fMAP aspect ratio, tibial mediolateral (tML), middle anteroposterior (tAP), medial anteroposterior (tMAP), and lateral anteroposterior (tLAP) tML/tMAP aspect ratio to determine the morphologic differences between OA and normal knees.

**Results:**

The average fMCW and tMAP dimensions of OA knees were larger than those of normal knees in both male and female (*p* <0.05). The fMAP/fML aspect ratio and tMAP/tML aspect ratio were also significantly different in both sexs (*p* <0.05). OA knees have an oval-shaped distal femur with a wider ML length and more spherical-shaped proximal tibiae with relatively narrow ML dimensions.

**Conclusions:**

The study revealed the morphological differences in fMCW, tMAP, fMAP/fML and tMAP/tML between OA and normal knees in both males and females. These findings may provide guidelines that can be used to design better knee implants that are more size-matched for OA knees.

## Background

An appropriate prosthesis size that matches the resected bony surfaces is considered a crucial factor for success in total knee arthroplasty (TKA) [1, 2]. If the prosthesis underhangs the resected surface of the bone, it may cause early subsidence and loosening of the prosthesis, whereas an overhang may cause residual pain, poor knee flexion, and decreased functional results [3, 4]. Thus, it is important to maximize coverage of the knee component on the resected bony surface to ensure a good clinical result and long-term survivorship of the prosthesis [5, 6]. To design a proper knee component, many researchers have measured the resected surface of normal knees from imaging data [7, 8], while others have analyzed anthropometric features of diseased knees during TKA [9, 10]. It is unclear whether there are morphometric differences in the resected bony surfaces between diseased and normal knees.

Generally, most knees that undergo TKA are deformed and shaped differently than healthy knees. This suggests that the design of the prosthesis should be based on data from diseased knees [11]. However, most of the currently available TKA prostheses are designed based on the anthropomorphic features of normal knees [12]. Such prostheses may not necessarily provide the best fit for TKA candidates. Osteoarthritis accounted for more than 90% of the patients who underwent primary TKA [13, 14]. To the best of our knowledge, no morphometric differences in the resected distal femur and proximal tibia surface between the OA and normal knees have been compared. The aim of this study, therefore, was to measure the morphometric features of the resected distal femur and proximal tibia surface to determine whether there are morphometric differences between OA and normal knees.

## Methods

This study was performed with the informed consent of each subject and approved by the institutional review board of Shaanxi Provincial People’s Hospital. In this study, the morphology of 94 (49 males and 45 females) OA knees that were candidates for TKA and 95 (48 males and 47 females) normal knees after anterior cruciate ligament construction or knee trauma without fracture, congenital anomalies or pathological deformities around the knee joint were recorded from June 2017 to April 2018. According to the Kellgren and Lawrence classification, all OA knees had radiographic evidence of grade III-IV osteoarthritis. There were 12 grade III and 37 grade IV in males and 9 grade III and 36 grade IV in females.

Computer tomography (CT) imaging was performed using a helical CT scanner imaging machine (120 kVp, 200 mA, Somatom Sensation, Siemens Health care, Germany). The subjects were placed in the supine position on the scanner with knees in the full extended position and their patella facing toward the ceiling. The scanning procedure was performed to acquire 1.0 mm CT slices (image size: 512×512 pixels). In the CT scan, a 15 cm femur and tibia diaphysis were included. The images of the knees were segmented using a region-growing method to construct 3D bony models by Mimics 17.0 software (Belgium, Materialise). The measurements were performed using Geomagic Studio 14.0 software (USA, Raindrop).

**Fig. 1:**
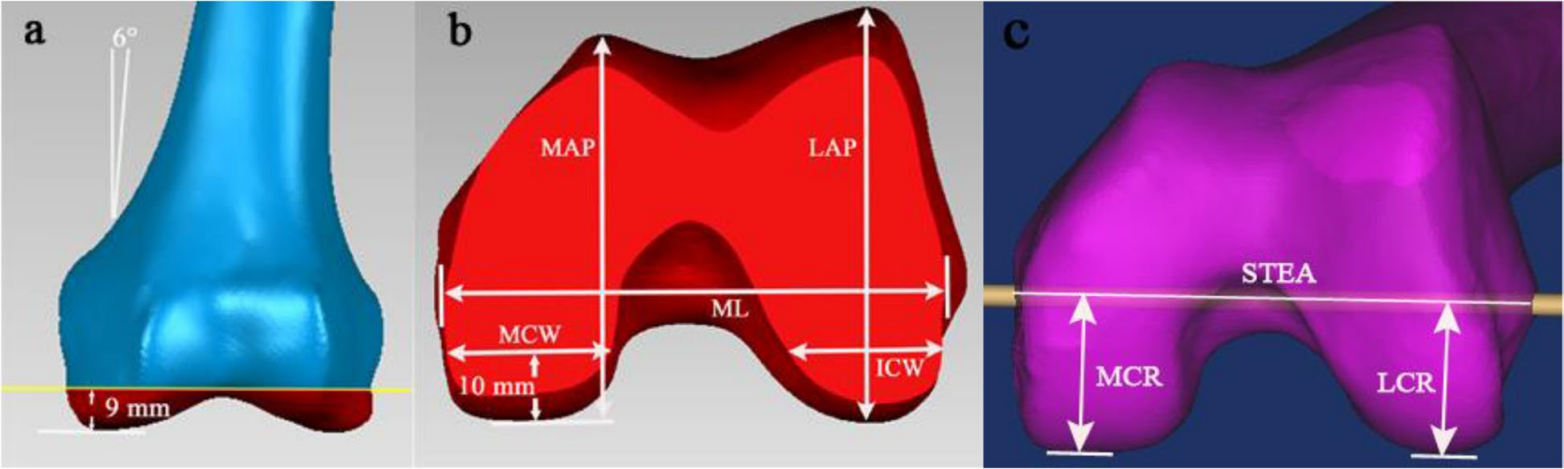
Distal femur resection and measurement on CT images. **a** Resection method of distal femur. **b** Measurements of resected femoral surfaces. **c** Measurements of femoral posterior condyle curvature radii

A point was marked in the center of the femoral intramedullary canal 12 cm from the distal femoral joint surface. A line connecting this point and the point 5 mm anterior to the intercondylar notch apex was defined as the femoral anatomic axis. The distal femur was cut 9 mm above the lowest point of the medial condyle with 6° valgus to the anatomical axis (Fig. 1 a). A line connecting the medial sulcus (the insertion point of the deep fibers of the medial collateral ligament )[15] of the medial epicondyle and the lateral epicondylar prominence was defined as the surgical transepicondylar axis (STEA). The femoral mediolateral (fML) dimension was defined as the longest ML length of the distal cut femur surface; this line paralleled the STEA. The femoral lateral anteroposterior (fLAP) and medial anteroposterior (fMAP) dimensions were defined as the longest line drawn perpendicular to the fML between the most posterior condylar and the anterior trochlear point from the lateral and medial condyle of the femur. The medial and lateral condyle widths were measured 10 mm above the lowest point of the medial posterior condyles to simulate the optimal cutting thickness (Fig. 1 b). The femoral medial posterior condyle curvature radii (fMCR) and lateral posterior condyle curvature radii (fLCR) were defined as the vertical distance between the STEA (functional flexion-extension axis) and the most posterior point of the medial and lateral posterior condyles, respectively (Fig. 1 c).

**Fig. 2:**
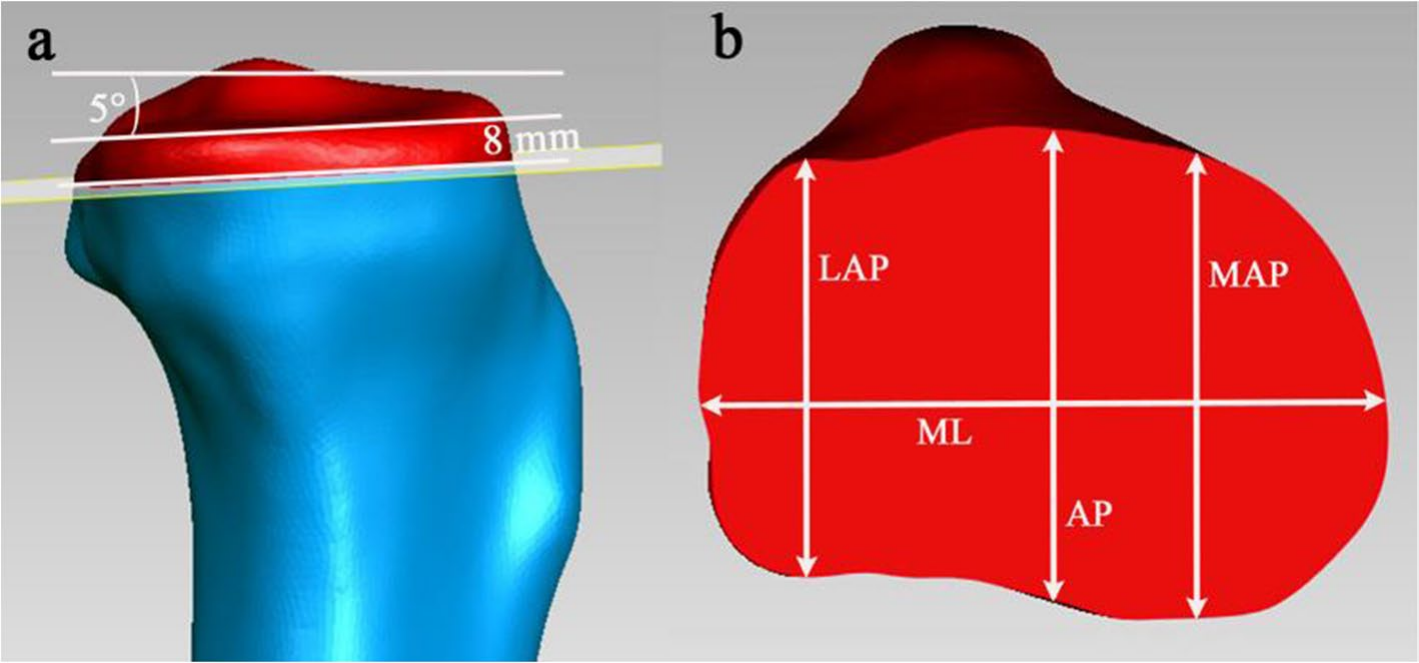
Proximal tibia resection and measurement on CT images. **a** Resection method of proximal tibia. **b** Measurements of resected tibial surfaces

A point was marked in the center of the tibial intramedullary canal 12 cm from the proximal tibial joint surface. A line connecting this point and the center of the tibial spines was defined as the tibial mechanical axis. The proximal tibial cut was performed perpendicular to the mechanical axis of the tibia, 8 mm below the lateral tibial plateau with 5° of posterior inclination (Fig. 2 a). The tibial mediolateral (tML) dimension was taken as the longest mediolateral length of the resected tibial surface. This line is parallel to the surgical epicondylar axis of the femur and formed by connecting the medial sulcus of the medial epicondyle and the lateral epicondylar prominence. The tibial middle anteroposterior (tAP) dimension was taken as the length of the line drawn perpendicular and passing through the midpoint of the tML line. The tibial lateral anteroposterior (tLAP) and medial anteroposterior (tMAP) dimensions were taken as the length of the line drawn perpendicular to the tML and passing through the posterior-most point of the lateral and medial tibial condyle (Fig. 2 b). The condylar aspect ratios of fMAP to fML (fMAP/fML) and the plateau aspect ratio of tMAP to tML (tMAP/tML) were calculated as described by Hitt [16].

### Statistical analysis

SPSS software 18.0 (SPSS, Chicago, IL) was used for statistical analysis. The mean and standard deviation of the measured dimensions were calculated. A Kolmogorov–Smirnov test for normality was performed, and the data were found to be normally distributed. Independent sample t-tests were used to determine the significance of morphological differences between OA and normal knees. To determine the influence of height on the knee joint dimensions, all data were additionally corrected for body height. The differences were regarded as significant when *p* <0.05.

## Results

OA subjects were older, lower and had larger HKA angles than normal knee subjects. There was a significant difference in the body weight of female subjects (p<0.01). The demographic characteristics of the subjects are summarized in Table 1.

**Table 1:** The demographic data of subjects

Parameter	Male			Female		
	OA	Normal	*p*	OA	Normal	*p*
Age (year)	64.6 ± 6.2	28.8 ± 5.7	<0.01	65.5 ± 5.9	31.1 ± 7.0	<0.01
Body weight (kg)	75.7 ± 9.8	75.3 ± 10.5	0.886	67.0 ± 8.4	66.6 ± 9.8	0.815
Height (cm)	170.2 ± 7.3	175.1 ± 6.5	<0.01	160.5± 5.8	164.7 ± 6.2	<0.01
HKA(°)	6.8 ± 3.2	0.8 ± 1.9	<0.01	8.3 ± 2.1	1.4 ± 2.3°	<0.01

On the basis of the numbers available, the average fMCW dimensions (28.9±2.8 mm for males and 27.3±2.4 mm for females) of OA knees were larger than the dimensions (27.0±2.9 mm for males and 25.4±2.3 mm for females) of normal knees (*p*<0.01). The condylar aspect ratio (fMAP/fML) was also significantly different (p<0.05). After correcting for body height, there was a significant difference in fMCW and fMAP/fML in both males and females (*p*<0.05). Morphological differences found between the OA and normal groups in terms of distal femur dimensions are summarized in Table 2.

**Table 2:** Distal femur dimensions in OA and normal knees (mm)

Parameter	Male				Female			
	OA	Normal	*p*	Corrected *p*	OA	Normal	*p*	Corrected *p*
fML	76.8±3.5	76.3±3.2	0.493	0.121	71.4±4.3	70.7±3.2	0.401	0.136
fMAP	64.0±3.7	64.7±2.7	0.267	0.262	59.8±3.2	60.5±3.6	0.320	0.188
fLAP	66.7±3.8	67.3±3.3	0.431	0.193	61.4±3.1	61.7±4.8	0.790	0.239
fMCW	28.9±2.8	27.3±2.4	0.018	<0.01	27.0±2.9	25.4±2.3	<0.01	<0.01
fLCW	26.8±2.9	26.4±2.7	0.299	0.062	24.6±2.1	24.2±2.0	0.358	0.103
fMCR	23.7±2.1	24.2±2.3	0.392	0.061	21.6±2.0	22.2±2.2	0.296	0.290
fLCR	22.1±2.1	22.3±1.8	0.610	0.293	20.5±2.2	20.9±2.2	0.356	0.537
fMAP/fML	0.83±0.03	0.85±0.03	0.028	0.028	0.84±0.04	0.86±0.03	0.027	0.028

The average tMAP dimensions and plateau aspect ratio (tMAP/tML) showed significant differences between OA and normal knees in both males and females (p<0.05). After correcting for body height, there was also a significant difference in tMAP and fMAP/fML in both sexes (p<0.05) (Table 3).

**Table 3:** Proximal tibia dimensions in OA and normal knees (mm)

Parameter	Male				Female			
	OA	Normal	*p*	*Corrected p*	OA	Normal	*p*	*Corrected p*
tML	78.6±3.5	78.8±3.2	0.836	0.148	72.2±3.2	71.9±3.1	0.651	0.135
tMAP	55.0±3.1	53.7±2.1	0.017	<0.01	50.6±2.7	48.9±2.3	0.002	<0.01
tAP	52.7±3.2	52.3±3.0	0.371	0.054	47.7±2.7	47.4±2.2	0.570	0.116
tLAP	50.7±3.6	50.1±3.3	0.382	0.067	45.6±3.0	45.3±2.6	0.529	0.087
tMAP/fML	0.70±0.03	0.68±0.02	<0.01	<0.01	0.70±0.02	0.68±0.03	0.027	0.027

## Discussion

In this study, we measured the morphology of the resected distal femur and proximal tibia surfaces of OA and normal knees. The major findings were that the fMCW and tMAP dimensions in OA subjects were significantly larger than those of normal knees. In a study by Puthumanapully et al., the authors found that varus knees had larger femur dimensions of the medial condyle than normal knees [17]. The morphological differences in the medial condyle between OA and normal knees may be explained by the pathological changes in OA knees. Most OA knees of TKA candidates had varus deformity, and the medial condyle experienced destruction and remodeling in response to larger loads during gait [18], which could eventually result in bony structural changes in the medial condyle of OA knees. In addition, there was a difference in the distal femur condyle and proximal tibia plateau aspect ratio between OA and normal knees. OA knees were found to have an oval-shaped distal femur with a wider ML length and more spherical-shaped proximal tibiae with relatively narrow ML dimensions.

Optimal coverage of the component to the resected bony surface is essential for long-term good outcomes after TKA. If the implant mismatches the resected bone surface, there will be undersizing or overhang, which could result in worse clinical outcomes [19]. Thus, it is critical to design a size-matching component for TKA candidates according to knee morphology. Various morphological studies of resected bony surfaces from normal [7, 20] or OA knees [9, 21] have been conducted to provide data for proper size matching. Cheng et al. [11] suggested that the design of the knee component should be based on data from diseased knees rather than normal knees. To date, no studies have examined the morphological differences of resected femoral and tibial surfaces between diseased and normal knees to determine which morphological data are more suitable to use to design proper components.

Many studies have reported on the measurements of resected proximal tibia surfaces in Asian knees. Cheng et al. [7] reported mean tML, tAP, tMAP, and tLAP values were 76.4 mm, 51.3 mm, 53.3 mm, 47.7 mm for male and 68.8 mm, 45.7 mm, 47.5 mm, 42.4 mm for female in 172 Chinese normal tibias by CT imaging. Kwak et al. [22] studied 200 normal cadaver tibias and determined that the tML, tAP, tMAP, and tLAP values were 76.1 mm, 48.2 mm, 48.8 mm, and 44.6 mm for males and 67.6 mm, 43.2 mm, 43.5 mm, and 39.8 mm for females, respectively, on CT imaging. Karimi et al. [5] studied 132 normal tibias from the Iran population on MRI scans and determined that the tML, tAP, tMAP, and tLAP values were 77.8 mm, 48.8 mm, 53.1 mm, and 52.0 mm for males and 66.5 mm, 43.1 mm, 45.5 mm, and 43.7 mm for females. The results in our Chinese subjects were slightly larger than those in the overall Asian population, which might be due to the difference in imaging techniques and the difference in the heights of the individuals in the study group. In addition, the depth of the resection affects the sizing of the resected bony surface. The depth of the proximal tibia resection in our study at a thickness of 8 mm below the lateral tibial plateau was higher than the 10 mm [5] or lower than the 6 mm [22] depth used in other studies.

Several researchers have studied the dimensions of distal femurs in Asian populations. Cheng et al. [7] reported the mean fML and fLAP values on CT to be 74.4 mm and 66.6 mm for males and 66.8 mm and 61.0 mm for females, respectively, in normal Chinese femurs. Lim et al. [20] showed that femoral fML, fMAP and fLAP dimensions were 81.5 mm, 62.7 mm, 59.0 mm for males and 76.7 mm, 56.8 mm, 58.4 mm for females in a Korean population using MRI. Urabe et al. [23] studied the distal femur using CT imaging in a Japanese population and reported fML, fMCW and fLCW dimensions of 70.6 mm, 30.1 mm and 24.8 mm in OA subjects. Vaidya et al. [24] used CT to show that femoral ML and LAP dimensions were 68.8 mm and 56.6 mm in males and 64.1 mm and 56.8 mm in females in Indian OA knees. Our results showed minor differences between these Asian populations. which might be due to the difference between the heights of the study groups. Charlton et al. [25] reported a significant difference in the femoral bicondylar width between short and tall subjects, with taller subjects having larger values.

To date, many studies have confirmed knee anatomic differences in Caucasian and Asian populations [26, 27]. However, nearly all existing TKA components were designed based on the anatomy of Caucasian populations and are not suitable for Asian patients [7, 28]. In the clinic, Iorio et al. followed (9 vs. 6.6 years) American and Japanese patients after primary TKA and showed that American patients required significantly larger implants than Japanese patients. The authors also found that Japanese patients had significantly less postoperative range of motion (93.7 vs. 106.6°) and a higher revision rate (4.1% vs. 2.6%) than American patients [29]. Anatomy studies and clinical outcomes all demonstrated that ethnic differences should be considered when designing proper TKA components for Asian populations.

We acknowledge that this study included a limited number of subjects and may not adequately reflect the features of OA and normal knees. If a larger sample size was included in the study, other significant differences may be noticed. We are also aware that only one bone resection level was measured. However, resection depth varies according to the stage of disease during TKA. In the future, we will report data for a larger sample size and measure the depth at different resection levels.

## Conclusion

In summary, our study demonstrated that the fMCW, tMAP dimensions, and MAP/ML aspect ratio of the distal femur condylar and proximal tibia plateau of resected knee surfaces were indeed significantly different in OA knees than in normal knees. As a result, we believe that the shape variations of the OA knees and normal subjects should be a concern when designing components for TKA candidates.

## Data Availability

The datasets used and/or analyzed during the present study are available from the corresponding author on reasonable request.

## Abbreviations

OA: Osteoarthritis
tML: Tibial mediolateral
tAP: Tibial middle anteroposterior
tMAP: Tibial medial anteroposterior
tLAP: Tibial lateral anteroposterior
fML: Femoral mediolateral
fMAP: Femoral medial anteroposterior
fLAP: Femoral lateral anteroposterior
fMCW: Femoral medial condylar width
fLCW: Femoral lateral condylar width
fMCR: Femoral medial posterior condyle curvature radii
fLCR: Femoral lateral posterior condyle curvature radii
TKA: Total knee arthroplasty
CT: Computer tomography
STEA: Surgical transepicondylar axis
BMI: Body mass index
HKA: Hip knee ankle
